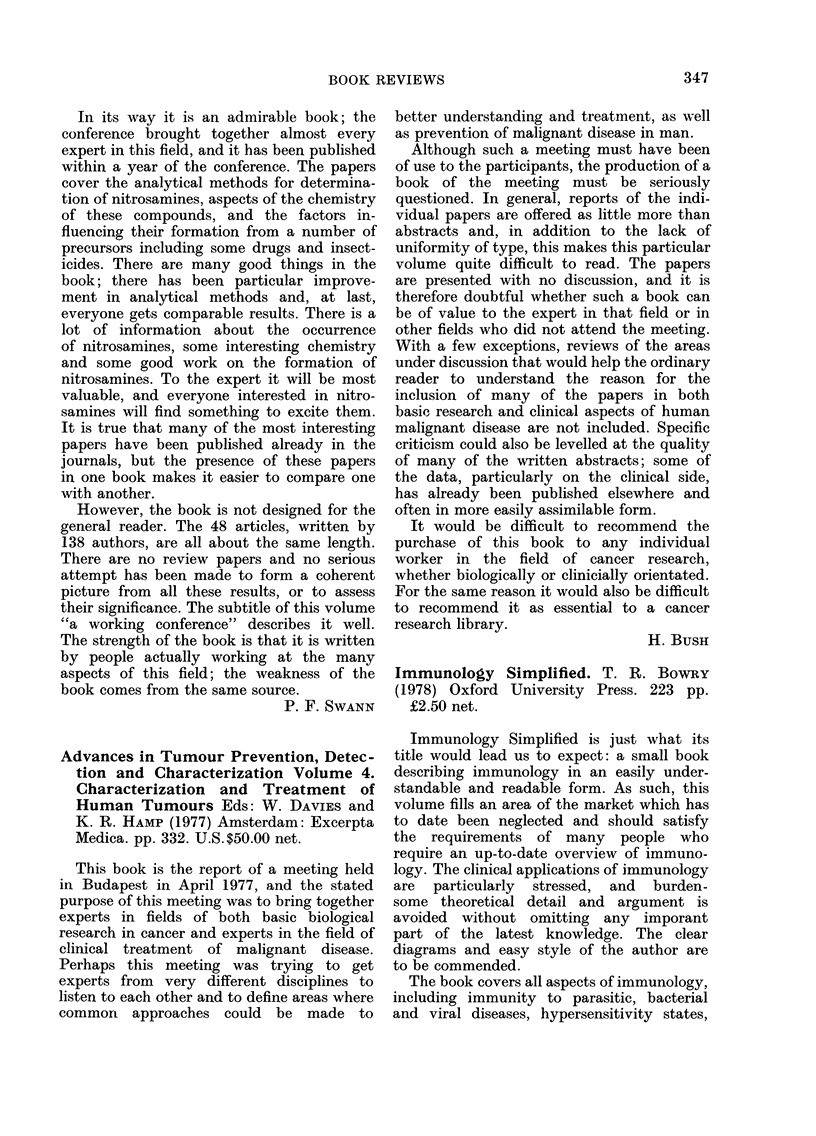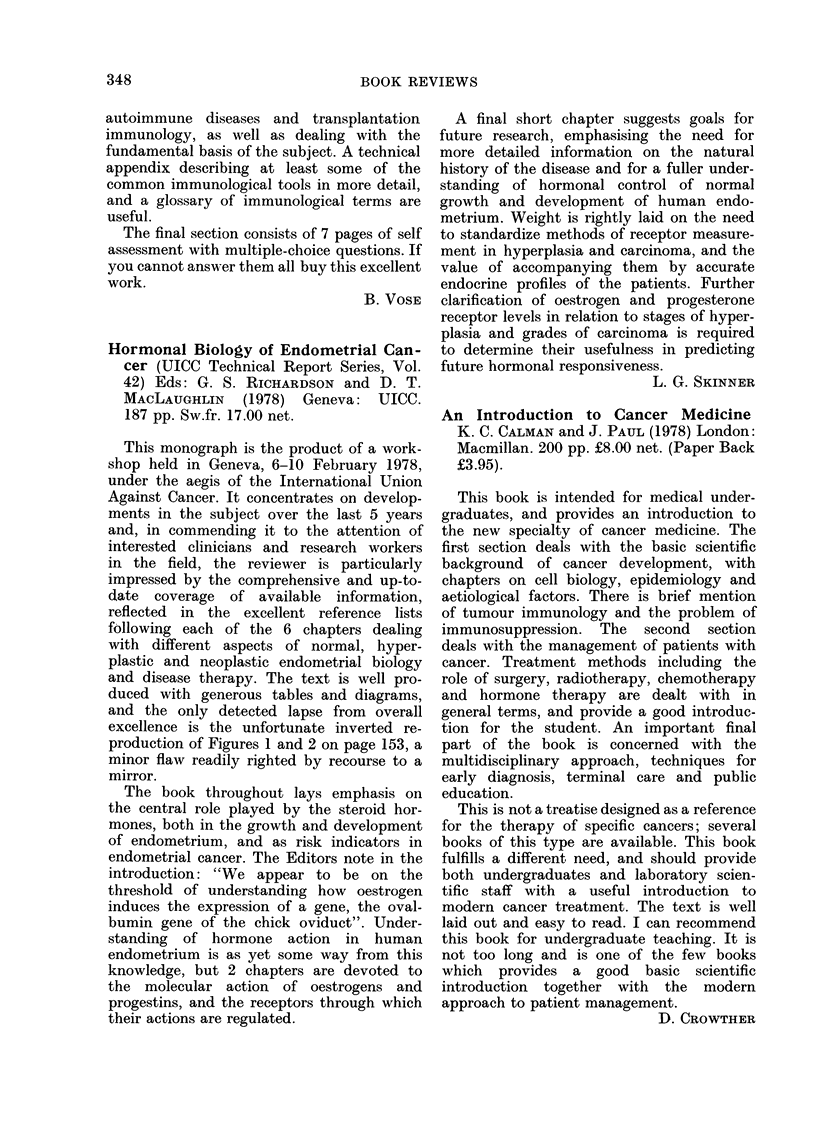# Immunology Simplified

**Published:** 1979-03

**Authors:** B. Vose


**Immunology Simplified.** T. R. Bowry (1978) Oxford University Press. 223 pp. £2.50 net.

Immunology Simplified is just what its title would lead us to expect: a small book describing immunology in an easily understandable and readable form. As such, this volume fills an area of the market which has to date been neglected and should satisfy the requirements of many people who require an up-to-date overview of immunology. The clinical applications of immunology are particularly stressed, and burdensome theoretical detail and argument is avoided without omitting any imporant part of the latest knowledge. The clear diagrams and easy style of the author are to be commended.

The book covers all aspects of immunology, including immunity to parasitic, bacterial and viral diseases, hypersensitivity states, autoimmune diseases and transplantation immunology, as well as dealing with the fundamental basis of the subject. A technical appendix describing at least some of the common immunological tools in more detail, and a glossary of immunological terms are useful.

The final section consists of 7 pages of self assessment with multiple-choice questions. If you cannot answer them all buy this excellent work.

B. VOSE